# Iron Chelation Properties of Green Tea Epigallocatechin-3-Gallate (EGCG) in Colorectal Cancer Cells: Analysis on Tfr/Fth Regulations and Molecular Docking

**DOI:** 10.1155/2020/7958041

**Published:** 2020-03-21

**Authors:** Zarith Nameyrra Md Nesran, Nurul Husna Shafie, Siti Farah Md Tohid, Mohd Esa Norhaizan, Amin Ismail

**Affiliations:** ^1^Department of Nutrition and Dietetics, Universiti Putra Malaysia, 43400 Serdang, Malaysia; ^2^Laboratory of UPM-MAKNA Cancer Research, Institute of Bioscience UPM, 43400 Serdang, Malaysia; ^3^Department of Biomedical Sciences, Universiti Putra Malaysia, 43400 Serdang, Malaysia

## Abstract

In many studies, green tea epigallocatechin-3-gallate (EGCG) has already shown its therapeutic effects in colorectal cancer cells (CRC). However, its mechanism of actions in CRC is poorly elucidated. Hence, this study attempts to elucidate the mechanism of actions of green tea ECGG via iron chelation activity in CRC. In order to investigate this property, HT-29 cell lines (CRC) were treated with EGCG for 24 h, 48 h, and 72 h. From western blot analysis, EGCG had upregulated transferrin receptor (TfR) protein and downregulated Ferritin-H (FtH) protein indicating that iron chelation activity has occurred in CRC. Meanwhile, the molecular docking study demonstrated that EGCG is able to strongly interact the ferritin protein with a high binding affinity (−7.3 kcal/mol) via strong hydrogen bindings to glutamic acid 64 and lysine 71; two moderate hydrogen bindings to asparagine 74 and a hydrophobic interaction to the hydrophobic pocket of lysine 71. The strong interaction predicted between EGCG to ferritin may lead to inhibition of ferritin by EGCG, thus supporting the downregulation of FtH observed in in vitro studies. Molecular docking study of TfR to EGCG cannot be modulated based on the in vitro results. In conclusion, EGCG possesses iron chelator property in CRC and this potential could be further exploited for CRC treatment.

## 1. Introduction

Since 500 000 years, China and Japan, in particular, have been consuming green tea on a daily basis to the extent of making green tea as health treatment purpose [1]. The green tea, also known scientifically as *Camellia sinensis* L, is rooted in tea plants. Tea leaves contain high polyphenols [2], and green tea has a significantly greater amount of polyphenols content [3] in comparison with black tea and oolong tea. It is these polyphenols, primarily catechins, that are responsible for the green tea health effects [2, 4].

The tea catechins are classified under the flavonoids family that has benzene rings known as A and B rings. Additionally, the catechin structure possesses the C ring (dihydropyran heterocycle) that has a hydroxyl group attached to carbon number 3. The A- and B-rings are similar to resorcinol and catechol moiety, respectively [5].

**Figure 1:**
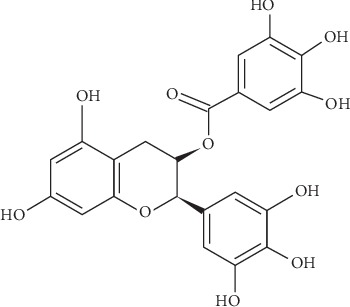
The structure of EGCG.

Epigallocatechin-3-gallate or shortly known as EGCG is the most abundant catechin and accounts for 50%–75% of the total amount of catechins. Furthermore, EGCG constitutes approximately 40% in the polyphenols group itself. Also, EGCG appears to be the most effective constituent of green tea [6].  Figure 1 shows the chemical structure of EGCG.

EGCG has demonstrated as the most effective polyphenol in green tea as a chemopreventive agent. It has antiproliferative effects over colorectal cancer cell lines SW-480 and HCT-116 by induction of apoptosis [7]. The same antiproliferative effect of EGCG was observed on HCT-116 [8] and HT-29 [9]. Furthermore, the administration of EGCG was able to suppress liver metastasis in colorectal cancer [8]. The capability of EGCG as a suppressor was also observed in colorectal cancer stem cells via the Wnt/*β*-catenin pathway [10].

Iron is an essential element for normal cellular function. However, excessive iron is very likeable by cancer cells as these irons lead to cancer progression. Thus, it is very important to remove these excessive irons from the cancer cells so that the tumor-related progression can be stunted with the lack or absence of iron [11, 12]. Generally, iron chelators have been explored as a cancer chemopreventive and chemotherapeutic agent [13].

Iron acquisition is mediated by the transferrin receptor (TfR), a membrane protein that internalizes differic transferrin through receptor-mediated endocytosis. TfR expression is strongly influenced by intracellular iron levels: iron deprivation activates TfR synthesis whereas the opposite occurs when irons are excessive in the cells [14]. Many evidences have demonstrated that treatment of cells with an iron chelator will increase TfR mRNA levels and TfR protein expression [15, 16]. Meanwhile, iron storage is mediated by ferritin protein, and ferritin expressions may be regulated by hypoxia and oxidative stress in cancer cells [17].

Desferrioxamine (DFO) drug was used as a positive control for iron chelation treatment in this study. DFO is known as an iron chelator which is able to cause apoptosis in colon cancer cells [18]. Furthermore, it is a membrane-permeable chelator, and these characteristics explain its iron deprivation activity in cancer cells [19]. The efficacy of DFO in minimalizing Fe-based cytotoxicity has made it as a potential candidate to be considered as an anticancer agent [20].

## 2. Materials and Methods

### 2.1. Source of EGCG and Its Preparation for the Treatment

The product number of EGCG is E4143. According to the manufacturer's product information (Sigma, Missouri USA), the source of this EGCG is from green tea. Its manufacturing processes include extraction with hot water and ethyl acetate from the green tea leaves. EGCG isolation from the organic phase was performed chromatographically, and its peak was detected at 280 nm. This EGCG product was stored in −20°C freezer throughout the project. All the treatments involved had used freshly prepared EGCG, minutes before the treatments started.

### 2.2. Cell Culture and Treatment

Cancer cell lines used throughout this study were human colorectal adenocarcinoma cell line, HT-29. The original source of this cell line was from the American Type Culture Collection (ATCC). The cells were grown in Dulbecco's Modified Eagle Medium (DMEM 1X high glucose), supplemented with 10% Fetal Bovine, 1% Sodium Pyruvate, and 1% Penicillin-Streptomycin antibiotics. The cells were maintained in an incubator with the settings of 5% CO_2_/95%O_2_ at 37°C. All the media supplementation were the products from Gibco, Thermo Fisher Scientific, USA. Treatments on HT-29 cell lines were performed with EGCG (IC_50_ concentration of 72 h–88.1 *μ*M) and DFO (250.0 *μ*M). HT-29 cells were first seeded on 30 mm tissue culture treated petri dish at 8 × 10^5^ for an overnight. Following 70% cell confluency, the cells were then treated with EGCG and DFO (Sigma-Aldrich, MO, USA) for 24 h, 48 h, and 72 h incubation period.

### 2.3. Protein Extraction

The medium was first removed, and the cells were washed thrice with cold PBS. After that, 1 mL of 1X RIPA lysis buffer containing 1% of protease inhibitor EDTA-free (Merck), and 1% of phosphatase inhibitor was added to each 30 mm petri dish containing the cells. The cells were then let incubated on ice for about 5 minutes. After that, the cells were gently scraped using a cell scraper and were collected into a 2 mL microtube. The cells were further incubated on ice for 15 minutes. After that, it was centrifuged at 10 000 rpm and at 4°C for 10 minutes. The supernatant was then collected and transferred into a labeled new 2 ml microtube. The quantification of proteins was immediately performed by using a BCA protein assay kit.

### 2.4. Western Blot

The protein samples were separated by SDS-PAGE at 110 V for 50 minutes. Transfer of samples from gel to a PVDF membrane was performed by wet transfer with transfer buffer (1X), loaded with ice packs, at 60 V for 2 hours. Next, membranes were dried for another hour at room temperature (RT) before being blocked with 5% BSA in TBS-T buffer and gently rocked on a shaker at RT. Incubation with the primary antibody was performed in TBS-T + 5% BSA for overnight in 4°C chiller with a dilution of 1 : 1000. The next day, each membrane was washed three times with TBS-T for 5 minutes each or total. The HRP-conjugated secondary antibody was diluted at 1 : 2500 in TBS-T + 5% BSA, and then the blots were incubated for 1 hour at RT and washed with TBS-T thrice.

Antibodies used throughout this study included TfR (Cat. #: 13208 Cell Signaling Technology, MA, USA), FtH (Cat. #: 3998 Cell Signaling Technology), which were purchased from Cell Signaling Technology, MA, USA; GAPDH (Cat. #: SC25778 Santa Cruz Biotechnology, TX, USA), and anti-rabbit IgG HRP-linked (Cat. #: 7074 Cell Signaling Technology, MA, USA).

### 2.5. Protein Detection

The Luminata™ Forte Western HRP substrate was used for protein detection. The substrate was added onto the membrane and was let incubated at RT for 2 minutes. Later, the membrane was viewed in a gel doc. Image Studio Lite Version 5.2 was used to quantify the band's intensity. All the bands were normalized with the loading control, GAPDH.

### 2.6. Molecular Docking

**Figure 2:**
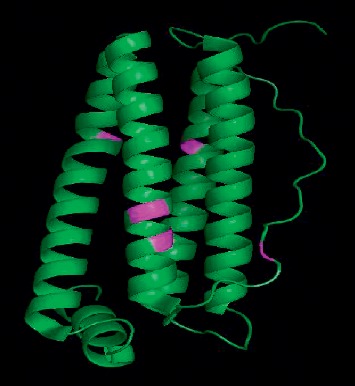
Crystal structure of human heavy chain ferritin obtained from PDB (PDB ID: 5N26). The protein binding sites determined from binding sites of cisplatin were coloured in magenta with a size of 22.6 kDa (1 polymer).

The ferritin receptor (FtH) was obtained from RSCB Protein Databank in “.pdb” format file with PDB ID of 5N26 at a resolution of 2.05 Å. This crystal structure was composed of one of the two chains indicated as the H chain with cisplatin intact to the binding sites of ferritin at four distinct binding sites (Figure 2).

**Figure 3:**
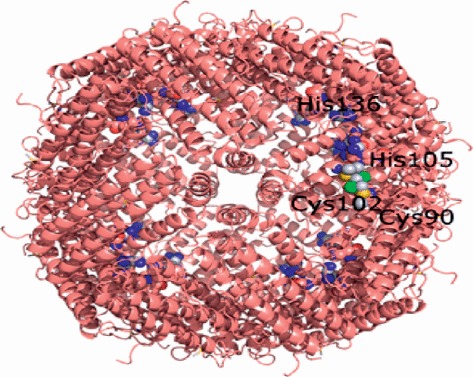
Full assembly of human heavy chain ferritin (size 543 kDa, 24 polymers) (reproduced with permission from Ferraro et al. [21]).

In physiological condition, ferritin is a globular protein complex consisting of 24 protein subunits forming a nanocage with multiple metal-protein interactions (Figure 3) [22].

The ferritin receptor was then visualized and prepared using AutoDock Tools 4.2 software [23]. This involved a number of stages. Firstly, unwanted ligand (cisplatin) was removed from the protein crystal structure, leaving only the target protein. Then, the crystal water was deleted from the structure as it could complicate the docking processes and calculation of binding affinities [24]. Next, hydrogen atoms were added to the structure. The crystal structure was then saved as “.pdb” files.

As for the ligand, EGCG, the structure was obtained from PubChem (https://pubchem.ncbi.nlm.nih.gov/) in “.sdf” format which was then converted to “.pdb” format file. Next, the protein and EGCG were prepared in PDBQT files format in order to run molecular docking job on AutoDock Vina. At this stage, Gasteiger partial charges were assigned on protein after merging nonpolar hydrogens, and the protein was kept rigid. The EGCG ligand was applied with torsion by rotating all rotatable bonds.

For docking, ECGC was docked to the binding sites of ferritin using AutoDock Vina software [25] with grid box coordinates (*x* = 72 Å; *y* = 36 Å; *z* = 84 Å, centred on ligand with *x*, *y* and *z* coordinates of 10.147, −29.237 and −52.564, respectively). Grid box coordinates were determined by referring to ferritin's active binding sites residues to cisplatin which were obtained from high-resolution X-ray crystallography: His105, Lys68, His136, Cys90, and Cys102 [21]. The binding interaction of ferritin with EGCG predicted by AutoDock Vina was further analysed using PyMol (the PyMOL Molecular Graphics System, Version 2.0 Schrödinger, LLC.) and ProteinsPlus (PoseView) [26] software.

### 2.7. Statistical Analysis

The protein expression results were analyzed using GraphPad software (San Diego, CA, USA). The independent *t*-test was applied to compare the differences in the mean between treated samples and control samples. Differences were only considered as statistically significant when the *P* value was <0.05.

## 3. Results and Discussion

### 3.1. EGCG Is a Potential Iron Chelator in Colorectal Cancer Cells (HT-29)

**Figure 4:**
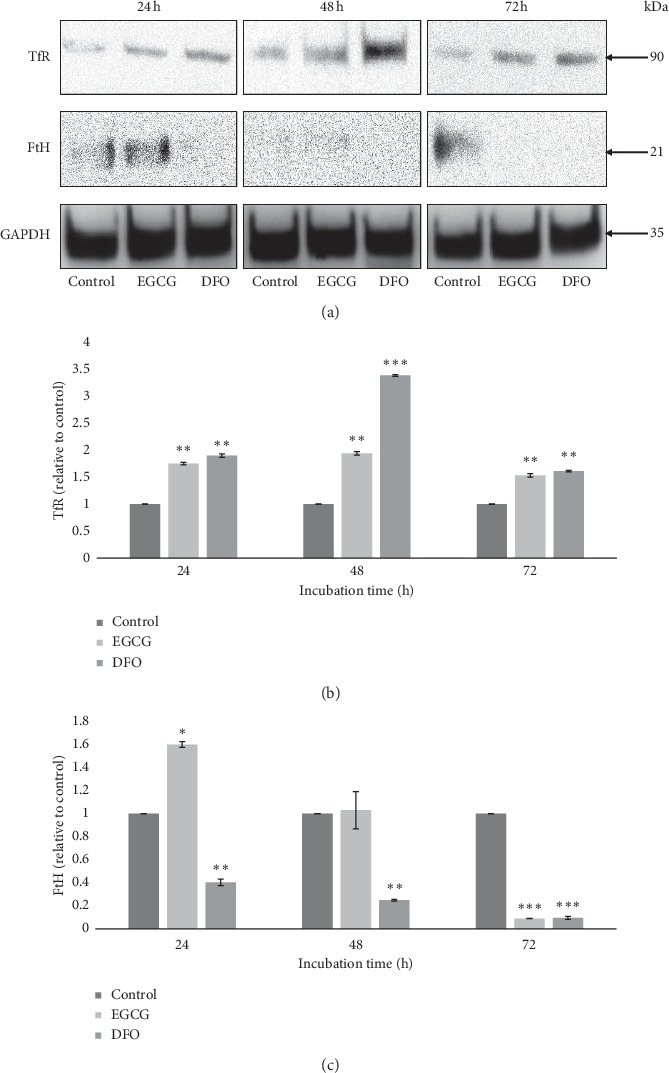
The expressions of TfR and FtH when given EGCG and DFO treatments. The densitometry results are from three independent experiments and are expressed as mean ± SEM normalized to GAPDH; ^*∗*^*p* < 0.05, ^*∗∗*^*p* < 0.01, ^*∗∗∗*^*p* < 0.001; relative to their respective control at each incubation time.

In this experiment, the treatment of EGCG had caused TfR being upregulated and FtH being downregulated, indicating that iron chelation activity had occurred in the HT-29.  Figure 4 shows TfR and FtH protein expressions and their protein densitometry results, respectively.

Treatment with EGCG showed that all the expressions of TfR were significantly upregulated (*p* < 0.01) at all incubation times; 24 h, 48 h, and 72 h (Figure 4). Basically, this experiment concluded that incubation time does not affect the significance of TfR expressions. Meanwhile, treatment with DFO also significantly increased the TfR after a 24 h (*p* < 0.01), 48 h (*p* < 0.001) and 72 h (*p* < 0.01) of incubations.

As shown in Figure 4, the FtH expression was significantly upregulated (*p* < 0.01) after EGCG treatment at 24 h and started to decrease but not significant (*p* > 0.05) after a 48 h incubation. However, after a 72 h of incubation, the expression of FtH was significantly downregulated (*p* < 0.001). Meanwhile, the DFO significantly decreased the expression of FtH after only a 24 h (*p* < 0.01), 48 h (*p* < 0.01) and 72 h (*p* < 0.001) of incubation in a time-dependent manner.

The results have shown that EGCG has upregulated the expression of TfR and downregulated the expression of FtH (Figure 4). These regulations in iron chelation-related proteins indicated that the activity of iron chelation led to iron depletion that occurred in colorectal cancer cells treated with EGCG.

Results from this study revealed that EGCG induced iron chelation activity in CRC cell lines, HT-29. Iron chelation occurrence in HT-29 was determined by the upregulation of the transferrin receptor (TfR). Generally, the expression of TfR is regulated via two modes: transcription and post-transcription [26]. Many evidences have demonstrated that treatment of cells with an iron chelator will increase TfR mRNA levels and TfR protein expression [15]. The inhibition of iron uptake from the TfR-Fe complexes is possible with the presence of chelators which in turn will inhibit tumor growth [27]. The main role of chelators is to chelate or form compound with metals such as iron (Fe) due to their affinity so that the binding of TfR-Fe complexes is made impossible. In the end, the goal of chelation therapy is to prevent the excessive amount of iron from accumulating in the cell [28]. The upregulation of TfR can be explained with the increased transcription of its specific mRNA that signals the synthesis of TfR should be enhanced upon iron chelation events in the cells [14]. Hence, EGCG is an iron chelator, can be said to possibly play an active role in enhancing TfR regulation in the HT-29 cells via increased transcriptional or posttranscriptional of specific mRNA. Iron chelation activity is a vital event in aiding cancer therapy [29] because the fate of cancer cells is very much affected by the disturbance in cellular iron homeostasis [13]. Furthermore, the risk of CRC increases with the excessive iron contents, either the source of iron coming from dietary intake or the already elevated iron levels in the body [30, 31].

The efficacy of a chelator depends on its chemical structure. Some related series of iron chelators have shown that their toxicity effects are associated with dipyridyl ring structure which modifies charge and lipophilic properties of the membrane. This alteration will grant chelators access to different cells containing iron [32]. In this study, EGCG has exhibited its properties as an iron chelator, hence it could be that its chemical structure which confers its chelating effects in CRC.

Khokhar and Owusu Apenten [33] listed three types of functional groups that are essential for binding with Fe^3^ which include (1) ortho dihydroxyl, (2) the addition of 5- and/or 3-OH to a C4 keto group, and (3) many numbers of OH group. EGCG is a compound with eight hydroxyl groups attached (at positions 3′, 4′ and 5′), thus EGCG can efficiently bind, hence chelate with iron [33]. Meanwhile, it was previously concluded that the metal chelation property is due to polyphenolic groups particularly at ring B [34]. It is with this chemistry position that EGCG was able to chelate with metals like cadmium (Cd), chromium (Cr), copper (Cu), and iron (Fe) [30].

Since colorectal cancer is linked with the iron overload in the body [31, 35], it is important to find an agent that can solve this challenge. Our findings have shown that EGCG is a potential agent that can chelate iron present in colorectal cancer. However, an extensive study is required to further validate the effect of increasing iron concentration in the cells on the iron chelation activity of EGCG. From the understanding of how iron chelation works, this study concludes that EGCG does trigger iron chelation activity in colorectal cancer cell lines, HT-29, hence caused iron depletion inside the cells. This conclusion is evident from the regulation of iron chelation-related proteins, TfR and FtH. Iron depletion is now also a major challenge for the CRC to keep surviving [11], making them get weaker and weaker as the EGCG treatment goes on. Eventually, the CRC will undergo apoptosis due to these threats.

### 3.2. Ferritin Was Identified as a Probable Target for EGCG

Computational molecular docking is a method that can predict experimental binding modes and affinities of small molecules [36]. This study used computational molecular docking to find the correct binding poses and binding affinities of EGCG to ferritin (Fth). Molecular docking is widely used to determine the binding of the potential chemical ligand with macromolecular targets, especially protein [37] which will eventually lead to protein inhibition and downregulation of protein expression as evidenced in the biological analysis [38]. We have shown earlier that EGCG markedly decreased the expression of FtH expression (Figure 4).

Unfortunately, regulation of EGCG to transferrin cannot be modulated *in silico* using the docking software, as the *in vitro* results revealed the upregulation of transferrin protein expression upon EGCG treatment which was not due to protein binding and inhibition.

The molecular docking result of EGCG to ferritin is outlined in Table 1.

Nine best binding modes of EGCG to ferritin were predicted by AutoDock Vina which were ranked from highest (the most negative) to the lowest binding affinity. Mode no 1 showed the highest binding affinity and was selected for further analysis.

ECGC was docked with the highest predicted binding affinity of −7.3 kcal/mol which is regarded as strong binding interaction.

**Figure 5:**
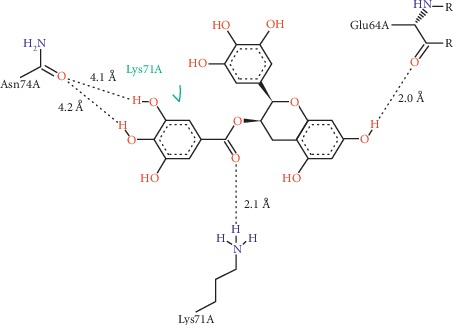
Binding interactions of FtH to EGCG as analysed by ProteinsPlus (Poseview) in two dimensions (2D). EGCG interacted with FtH residue via (i) hydrogen bindings (shown as dotted lines with distances measured in Armstrong, Å) and (ii) hydrophobic interaction (shown in green).

Detailed analysis of the binding in Figure 5 showed that the top binding affinity of ECGC to ferritin may be attributed to its formation of 4 hydrogen bindings and 1 hydrophobic interaction.

The hydroxyl group of ring B of EGCG at 3′ position can strongly bind to glutamic acid 64 at a distance of 2.0 Å via hydrogen bonding. At the same time, the ester (carboalkoxy) chain which linked ring A and ring B of EGCG exhibited strong hydrogen binding interaction with lysine 71 at a distance of 2.1 Å. The EGCG-ferritin binding was also contributed by weak hydrogen bindings between two hydroxyl groups of ECGC's ring A at 2- and 3- positions to asparagine 74 at 4.2 Å and 4.1 Å, respectively. Binding of EGCG to ferritin was also contributed by hydrophobic interaction between ring A with the hydrophobic region of lysine 71 of the protein. According to Jeffrey [39], hydrogen binding distances can be categorized as strong (2.2 Å–2.5 Å), moderate (2.5 Å–3.2 Å), and weak (3.2 Å–4.0 Å).

**Figure 6:**
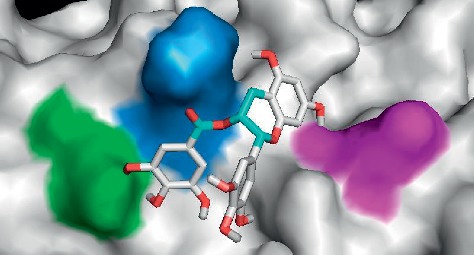
Position of EGCG in the hydrophobic pocket of ferritin. Ferritin has deep hydrophobic groove suitable for the binding of EGCG. Green = asparagine 74; blue = glutamic acid 64; and magenta = lysine 71.

Figure 6 showed the position of EGCG in the hydrophobic pocket of ferritin.

Hydrogen interaction is the preferred and most common interaction involved in the binding of a ligand and a protein (ligand-protein binding interaction) and is regarded as the most important specific interaction in biological recognition processes [40]. The study suggested that both strong and weak hydrogen bindings contributed significantly to the strong binding of ECGC to ferritin protein. The hydrophobic interaction with lysine 71 further strengthens the ligand-protein interaction. The strong EGCG-FtH binding predicted in the molecular docking study may explain the significant downregulation of expression of ferritin upon treatment with EGCG *in vitro*. This is supported by a number of studies which predicted the high binding ability (indicated as highly negative docking scores) of small molecules to protein of interest can be correlated to their protein inhibitory property, which resulted in downregulation of protein as observed in in vitro studies and comparable with known inhibitor for that particular protein [41–43]. Binding of ligand (EGCG) to a protein (FtH) may result in inhibition of the protein expression, which simultaneously will downregulate the expression of the protein.

## 4. Conclusions

It can be concluded that EGCG triggers iron chelation activity in colorectal cancer cell lines, HT-29. This study has discovered a new potential of EGCG which can be exploited for targeted colorectal cancer therapy.

## Figures and Tables

**Table 1:** Predicted binding poses of EGCG to ferritin.

Mode	Binding affinity (kcal/mol)
1	−7.3
2	−7.2
3	−7.1
4	−7.1
5	−7.1
6	−7.1
7	−7.1
8	−7.1
9	−7.0

## Data Availability

The molecular docking data used to support the findings of this study are available from the corresponding author upon request.
